# Acquired BRAFi Resistance Increases Melanoma Cell Sensitivity to Metabolic Targeting with Metformin

**DOI:** 10.3390/ijms27188088

**Published:** 2026-09-11

**Authors:** María Florencia Arbe, Gerardo Claudio Glikin, Liliana María Elena Finocchiaro, Marcela Solange Villaverde

**Affiliations:** Unidad de Transferencia Genética y Laboratorio de Metabolismo Tumoral, Área Investigación, Instituto de Oncología Ángel H. Roffo, Facultad de Medicina, Universidad de Buenos Aires, Av. San Martín 5481, Buenos Aires C1417DTB, Argentina

**Keywords:** melanoma, BRAFi, resistance, metformin, metabolism

## Abstract

The use of BRAF inhibitors (BRAFi) for the targeted inhibition of the MAPK pathway represents a cornerstone of treatment for BRAFV600E-mutant melanoma. However, the rapid emergence of drug resistance remains a major clinical challenge. Mounting evidence indicates that therapy resistance is driven by both phenotypic plasticity and metabolic reprogramming. This study aimed to investigate the molecular, phenotypic, and metabolic adaptations underlying acquired resistance to the BRAFi GSK2118436 in A375 melanoma cells. BRAFi-resistant cells (A375-R) displayed cross-resistance to additional BRAFi and MEK inhibitors and clear features of epithelial–mesenchymal transition (EMT), including decreased E-cadherin, increased vimentin, and inhibitor-dependent spheroid compaction. Among the multiple metabolic modulators evaluated, inhibitors of mitochondrial respiration—particularly metformin and antimycin A—selectively impaired viability, clonogenicity, sphere formation and 3D growth of resistant cells. Importantly, while simultaneous BRAFi–metformin combination caused mostly antagonist effects, a sequential regimen (BRAFi followed by metformin) elicited additive/synergistic cytotoxicity in both 2D and 3D models. Collectively, these findings show that BRAFi resistance is driven by coordinated EMT and metabolic rewiring toward OXPHOS dependency and suggest that sequential metabolic targeting is an effective strategy to overcome therapy resistance in melanoma.

## 1. Introduction

Cutaneous melanoma, one of the most aggressive skin cancers, is characterized by a high mutational burden and remarkable phenotypic plasticity. Among the genetic alterations driving melanoma progression, activating mutations in the *BRAF* gene—most frequently the V600E substitution—are detected in approximately 40–50% of cases and result in constitutive activation of the MAPK signaling pathway, promoting uncontrolled cell proliferation and survival [1]. In this context, the development of selective BRAF inhibitors (BRAFi), such as vemurafenib (PLX4032) and dabrafenib (GSK2118436), represented a major therapeutic breakthrough for patients with *BRAF*-mutant metastatic melanoma, significantly improving progression-free and overall survival [2,3,4]. However, despite high initial response rates, most patients relapse within months due to the emergence of acquired resistance. Resistance mechanisms include reactivation of MAPK signaling through secondary mutations or pathway bypass, activation of alternative survival pathways, and phenotypic switching [5,6,7].

Beyond genetic and signaling adaptations, increasing evidence indicates that melanoma cells undergo profound metabolic rewiring in response to antitumor strategies, especially during targeted therapies. Drug-resistant melanoma cells frequently display enhanced mitochondrial function and a shift toward oxidative phosphorylation (OXPHOS), which supports survival under therapeutic stress [4,8,9,10,11,12]. In this context, mitochondrial metabolism has emerged as a critical vulnerability in BRAFi-resistant melanoma. Indeed, increased reliance on OXPHOS has been shown to contribute directly to resistance against MAPK pathway inhibitors [4].

In this regard, metformin, a widely prescribed antidiabetic drug, is known to inhibit mitochondrial complex I, reduce ATP production, and activate AMP-activated protein kinase (AMPK), thereby indirectly suppressing mTOR signaling and anabolic metabolism. Thus, and due to its favorable safety profile and pleiotropic metabolic effects, metformin has attracted considerable interest as a potential anticancer agent. In melanoma, several preclinical studies have demonstrated that metformin can impair cell growth and survival, especially in metabolically adapted or therapy-resistant contexts [10,11,13,14,15].

In parallel, resistance to BRAFi has been associated with phenotypic transitions resembling epithelial–mesenchymal transition (EMT), characterized by changes in cell morphology, loss of epithelial markers, gain of mesenchymal traits, and increased cellular plasticity. These phenotypic changes may further reinforce metabolic flexibility and therapy resistance, suggesting a functional link between EMT, metabolic rewiring, and drug tolerance [16,17]. In thyroid cancer, BRAFV600E cells resistant to the BRAFi PLX4032 have been reported to display increased expression of mesenchymal EMT markers (vimentin, β-catenin, and CD44), together with enhanced migration and invasion processes [18]. In melanoma, BRAF mutations are frequent and known to regulate the invasive phenotype of tumor cells. Specifically, it has been demonstrated that BRAF signaling regulates the cadherin switch: in BRAFV600E melanoma cells, N-cadherin levels are high and E-cadherin levels are low, whereas BRAF silencing causes the opposite effect [19]. In addition, in melanoma cells resistant to vemurafenib, proteomic studies have revealed an increase in proteins associated with EMT, such as vimentin [20].

Given this background, in the present study, we hypothesized that acquisition of resistance to BRAFi could be accompanied by metabolic susceptibilities, particularly at mitochondrial level, thereby enhancing sensitivity to mitochondrial inhibition. To test this hypothesis, we established a dabrafenib (GSK2118436)-resistant melanoma cell line and systematically evaluated its molecular phenotypic and cytotoxic response to metabolic modulators such as metformin.

## 2. Results

### 2.1. Establishment of GSK2118436-Resistant A375 Cells

**Figure 1:**
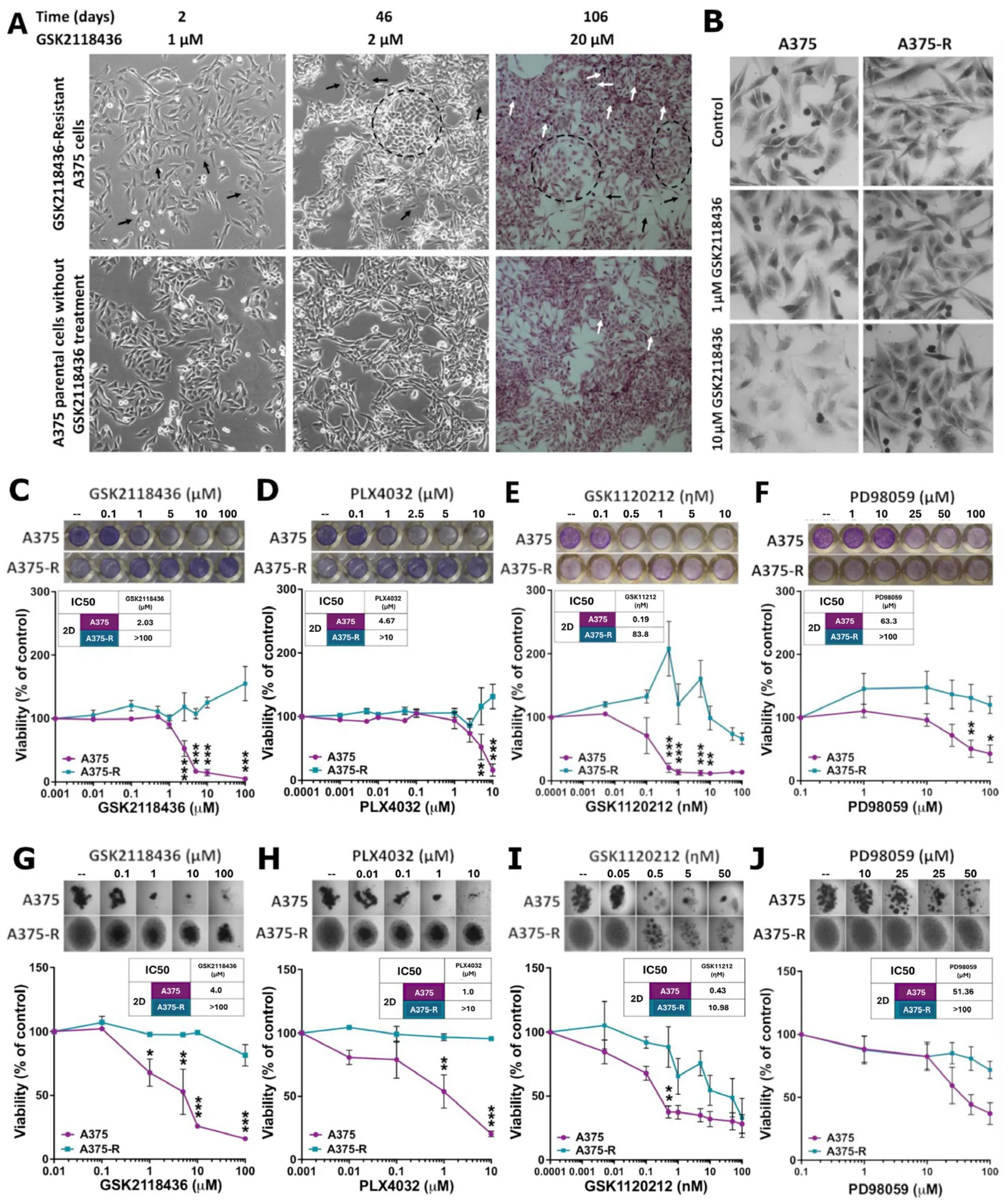
Effect of BRAF and MEK inhibitors on A375 and GSK2118436-resistant A375 (A375-R) cells. Representative images during A375-R development (**A**,**B**). A375 and A375-R cells were cultured under 2D (**C**–**F**) or 3D (**G**–**J**) conditions and then treated with increasing concentrations of the BRAF inhibitors GSK2118436 (0.001–100 µM) and PLX4032 (0.001–10 µM; (**C**,**D**,**G**,**H**)) or the MEK inhibitors PD98059 (0.001–100 nM; (**E**,**I**)) and GSK1120212 (0.01–100 μM; (**F**,**J**)). After 5 days (2D) or 14 days (3D) of culture, cell viability was evaluated using the acid phosphatase (APH) assay. Results are expressed as viability relative to control (mean ± s.e.m., *n* > 3). Viability data were analyzed using two-way ANOVA followed by Sidak’s multiple comparisons test (* *p* < 0.05, ** *p* < 0.01, *** *p* < 0.001) and fitted to a logarithmic inhibition curve with normalized response. IC_50_ values were obtained by curve analysis using GraphPad Prism 8.0.1 software.

To better understand the development of resistance to BRAF-targeted therapy, we first established a GSK2118436-resistant cell line by chronic exposure to this BRAFi. Treatment started with 1 μM GSK2118436. Cells increased their adhesion surface, developed irregular borders, and formed intercellular connections between distant, non-adjacent cells, altering cellular morphology (Figure 1A). This morphology was maintained for approximately 15 days, during which extensive cell death and decreased cell division were observed. Once the remaining cells began to recover their proliferative capacity, the concentration of the inhibitor was gradually increased. This cycle was repeated with successive passages. During the development of this resistant cell line, regions or *foci* of dense cellular proliferation were observed (black circles, Figure 1A), together with cells showing the previously described alterations (black arrows). Finally, after approximately four months of culture in the presence of the inhibitor, a GSK2118436-resistant cell line, which we named “A375-R”, was established (Figure 1A). As compared to the parental line, this cell line exhibited a higher number of cells in the supernatant (white arrows), retained regions with more compact cell morphology (black circles), and displayed cells with spindle-shaped morphology and extensive intercellular connections (black arrows). Resistant cells were maintained in culture under continuous selective pressure (20 μM GSK2118436). To validate that the phenotype observed was specifically associated with acquired drug resistance rather than long-term passaging, both A375-R cells and parental A375 cells were exposed to 1 µM and 10 µM GSK2118436 for 48 h. Under these conditions, control A375 cells displayed a morphology similar to that observed at the beginning of the selection process, characterized by reduced proliferation and altered adhesion, whereas A375-R cells preserved the resistant phenotype, maintaining their heterogeneous morphology with compact and spindle-shaped interconnected cells and a high proportion of non-adherent cells in the supernatant (Figure 1B).

### 2.2. Effect of BRAF and MEK Inhibitors on GSK2118436-Resistant A375 Cells

Once the A375-R cell line was established, the resistance generated against GSK2118436 was evaluated in comparison with the parental cells (A375). In addition, another BRAFi (PLX4032) was used to determine whether the resistance was specific to a single molecule or to BRAF inhibitors in general. Under 2D culture conditions, A375-R cells showed significantly lower sensitivity to GSK2118436 than the parental A375 cells starting at a concentration of 2.5 μM (*p* < 0.001; Figure 1C). Within the concentration range tested, no cytotoxic effect (i.e., no decrease in cell viability) was observed in A375-R cells, and the half-maximal inhibitory concentration (IC_50_) was 50-fold higher than that of the parental cells. Regarding the BRAFi PLX4032, A375-R cells also showed reduced sensitivity starting at 5 μM (*p* < 0.01; Figure 1D). In this case, a rightward shift in the response curve was observed for A375-R cells, with an IC_50_ more than twice that of A375 cells.

Although resistant cells remained viable under anchorage-independent growth, they failed to form 3D structures as effectively as A375 cells (Figure 1G,H A375-R cells without treatment). As in 2D cultures, A375-R cells exhibited significantly lower sensitivity to the inhibitor than A375 cells, as evidenced by a rightward shift in the curve data points (*p* < 0.05; Figure 1C,D), with an increase of more than 25-fold in the IC_50_ value relative to A375 cells, as well as by alterations in the morphology of the 3D structures.

The size of spheroids formed by A375 cells decreased in the presence of GSK2118436, whereas A375-R cells formed spheroids only in the presence of the inhibitor and in a concentration-dependent manner (Figure 1G). Similar results were obtained with the BRAFi PLX4032: when A375-R cells were exposed to PLX4032, their sensitivity was reduced compared to that of A375 cells starting at 1 μM (*p* < 0.01; Figure 1H), with an IC_50_ that was 10-fold higher, and cells in culture acquired a 3D structure only in the presence of the BRAFi.

Since BRAF kinase can phosphorylate and activate the kinases MEK1 and MEK2, it was of particular interest to investigate how sensitivity to MEK inhibitors (MEKi) was affected in A375-R cells. Under 2D culture conditions, A375-R cells exhibited reduced sensitivity to the two MEK inhibitors evaluated, GSK1120212 (trametinib) and PD98059, compared to A375 cells (Figure 1E,F). Specifically, for GSK1120212, the IC_50_ of A375-R cells was 441-fold higher (Figure 1E), whereas for PD98059 it was more than twice the IC_50_ of A375 cells (the IC_50_ of PD98059 for A375-R cells was not contained within the concentration range tested, Figure 1F). Under 3D culture conditions, A375-R cells treated with GSK1120212 were able to form structures compatible with multicellular spheroids, whereas those treated with PD98059 failed to generate such structures (Figure 1J). In both cases, A375-R cells exhibited lower sensitivity to MEK inhibitors, displaying an IC_50_ 25-fold higher for GSK1120212 (Figure 1I) and slightly more than two-fold higher for PD98059 (as in 2D culture, the IC_50_ for PD98059 in A375-R cells was not contained within the concentration range tested in 3D culture; Figure 1J).

### 2.3. Changes in EMT Markers

**Figure 2:**
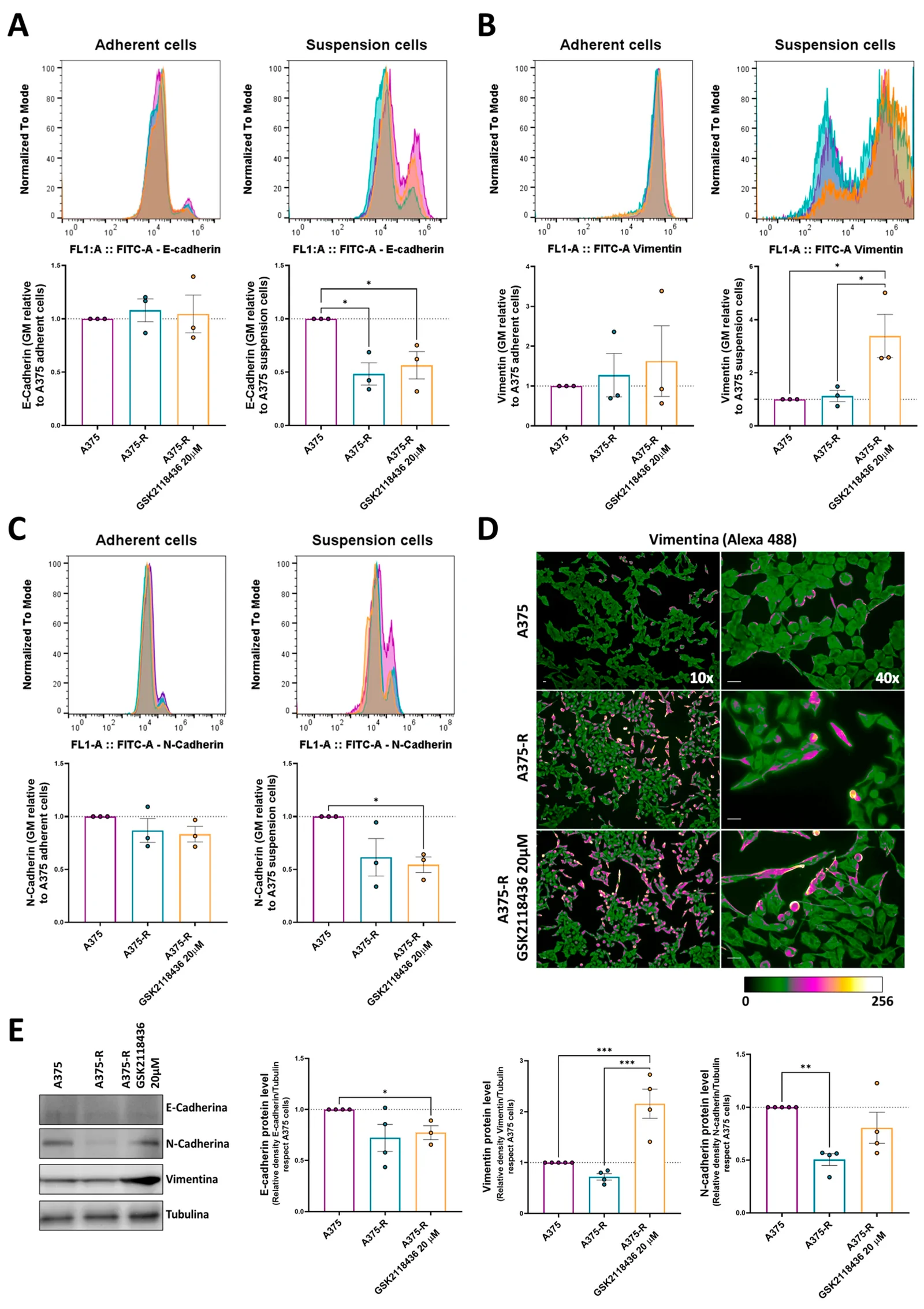
Changes in EMT expression patterns in GSK2118436-resistant melanoma cells (A375-R). Human melanoma A375 (purple) and A375-R cells, cultured in the presence (cyan) or absence (orange) of GSK2118436 (20 μM), were maintained for 48 h and subsequently separated into two populations: suspension or non-adherent (NAP) cells and adherent cells (AP) (see Section 4). E-cadherin (**A**), Vimentin (**B**) and N-cadherin (**C**) expression levels were then determined by indirect immunostaining followed by flow cytometry analysis. Vimentin subcellular distribution was evaluated by immunostaining and epifluorescence microscopy (**D**). Representative single-channel images (green) are shown using the Blue–Orange ICB look-up table, in which color differences reflect variations in fluorescence intensity (magnification: 100× and 400×). Scal bar: 20 μm. Results are expressed as the geometric mean fluorescence intensity relative to A375 cells (mean ± s.e.m.; *n* = 3), and representative histograms showing fluorescence intensity distributions are shown. Protein levels were assessed by Western blot analysis using total protein extracts (including both adherent and non-adherent cells) from each cell line, normalized to the loading control tubulin (**E**). Statistical analyses were performed using one-way ANOVA followed by Tukey’s multiple comparisons test (* *p* < 0.05, ** *p* < 0.01, ***, *p* < 0.001).

Considering the morphological changes observed, we next studied the expression of EMT markers, such as E-cadherin, whose presence is associated with a more epithelial phenotype, and vimentin, which is associated with a more mesenchymal phenotype. Since A375-R cells displayed a highly heterogeneous population, with a high percentage of non-attached cells, these markers were next evaluated in both the adherent population (AP) and the non-adherent population (NAP) (or suspension cells). Thus, A375 and A375-R cells, in the absence or presence of the BRAFi GSK2118436 (20 μM), were cultured for 48 h under 2D conditions, separated as AP and NAP populations and, subsequently, indirect immunolabeling was performed using monoclonal antibodies against E-cadherin and vimentin, and the results were analyzed by flow cytometry. No differences were detected in E-cadherin expression levels between A375-AP and A375-R-AP cells (Figure 2A); however, compared to A375-NAP cells, a significant decrease in E-cadherin expression was observed in A375-R-NAP cells both in the absence and presence of the BRAFi (*p* < 0.05). The histograms showing the distribution of labeling in suspension cells show that A375-NAP cells displayed a higher number of events with greater fluorescence intensity for E-cadherin than A375-R-NAP cells. These findings indicate that the non-adherent population of BRAFi-resistant cells exhibits a reduction in an epithelial-associated marker, suggesting a shift toward a less epithelial phenotype that is particularly evident in the NAP population.

Vimentin staining showed the opposite pattern: A375-R-AP cells exhibited a trend toward higher vimentin levels, and this pattern became significantly evident when comparing A375-R-NAP cells in the presence of the BRAFi with A375-NAP cells (*p* < 0.05; Figure 2B). This effect was also visualized by Western blot analysis using total protein extracts (AP and NAP) from both cell lines (Figure 2E). Taken together with the reduction in E-cadherin observed in the A375-R-NAP population, the increased vimentin expression provides additional evidence of a shift toward a more mesenchymal-associated phenotype in BRAFi-resistant cells, particularly under BRAFi exposure.

The distribution of vimentin was also evaluated by immunolabeling and epifluorescence microscopy. Representative images are shown on a single channel (green) and displayed using the Blue-Orange ICB look-up table, where color differences reflect differences in fluorescence intensity. Thus, fluorescence can be observed throughout each cell, with higher intensity in violet or orange regions (Figure 2D). Vimentin exhibited a differential distribution depending on the cell line: whereas A375 cells showed a more uniform distribution, A375-R cells, in both the absence and presence of the BRAFi, displayed a higher concentration of vimentin in the outermost cells of the monolayer. This altered spatial distribution of vimentin further suggests that acquisition of BRAFi resistance is accompanied not only by changes in EMT-associated marker expression but also by alterations in the organization of the cytoskeletal phenotype.

Overall, these results indicate that BRAFi-resistant A375 cells display alterations in EMT-associated features, with the most pronounced changes observed in the non-adherent population. The concomitant decrease in E-cadherin and increase in vimentin under BRAFi exposure are consistent with a more mesenchymal-associated phenotype. However, given the heterogeneous nature of the resistant population and the differential responses observed between AP and NAP cells, these findings are better interpreted as EMT-associated remodeling rather than evidence of a complete epithelial-to-mesenchymal transition.

### 2.4. Effect of Metformin and Other Metabolic Modulators

In this context, we hypothesized that blocking or regulating specific metabolic pathways could counteract the resistance phenomenon. For this reason, we decided to investigate the effect of metabolic modulators such as metformin and antimycin A on GSK2118436-resistant melanoma cells.

**Figure 3:**
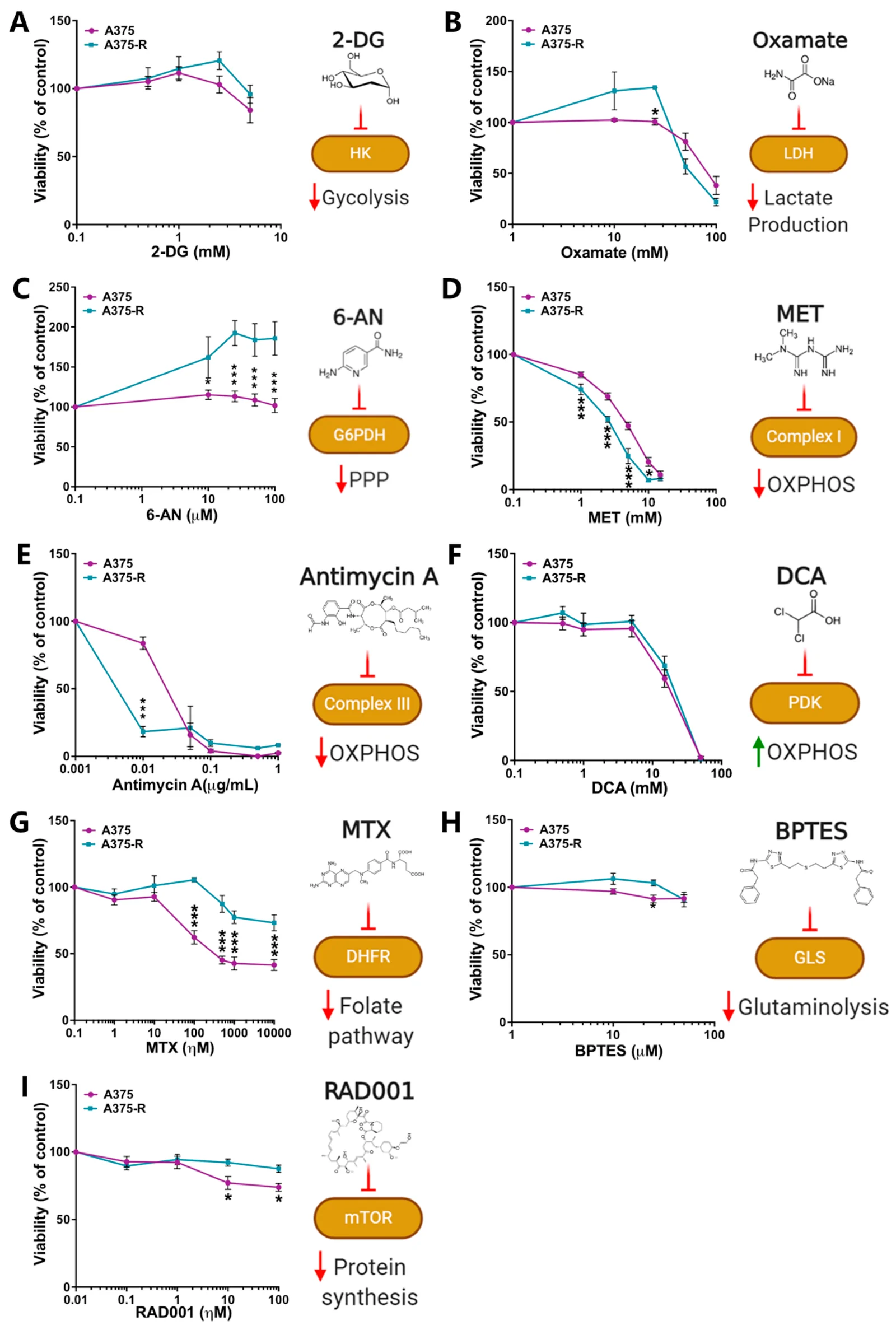
Cytotoxic effects of metabolic modulators in GSK2118436-resistant melanoma cells. A375 and A375-R melanoma cells were treated with increasing concentrations of 2-deoxyglucose (2-DG; (**A**)), oxamate (**B**), 6-aminonicotinamide (6-AN; (**C**)), metformin (MET; (**D**)), antimycin A (**E**), dichloroacetate (DCA; (**F**)), methotrexate (MTX; (**G**)), N,N′-[thiobis(2,1-ethanediyl-1,3,4-thiadiazole-5,2-diyl)]bis-benzeneacetamide (BPTES; (**H**)), and everolimus (RAD001; (**I**)). After 5 days of treatment, cell viability was assessed using the APH assay. Results are expressed as percentage of viability relative to untreated controls (mean ± s.e.m.; *n* > 3, * *p* < 0.05, *** *p* < 0.001). References: PPP: pentose phosphate pathway; OXPHOS: oxidative phosphorylation; HK: hexokinase; LDH: lactate dehydrogenase; G6PDH: glucose-6-phosphate dehydrogenase; PDK: pyruvate dehydrogenase kinase; DHFR: dihydrofolate reductase; GLS: glutaminase. T-bar arrows indicate inhibition, whereas standard arrows indicate a decrease (red) or an increase (green) in the corresponding level or activity.

To this end, the effects of nine different metabolic modulators (see Section 4 for details) were compared between A375 and A375-R cells. Cells were first exposed to increasing concentrations of the modulators, and, after five days of culture, cell viability was determined using the APH assay (Figure 3).

Seven out of the nine modulators, mainly metformin and antimycin A, showed differential responses (Figure 3). It is noteworthy that both metformin and antimycin A affect OXPHOS, which may suggest a mitochondrial role during resistance. In both cases, A375-R cells exhibited greater sensitivity to metformin (*p* < 0.001) and antimycin A (*p* < 0.001) than A375 cells (Figure 3). This effect was noticed from the leftward shift in the curves. and the changes in IC_50_ values (Table 1).

**Figure 4:**
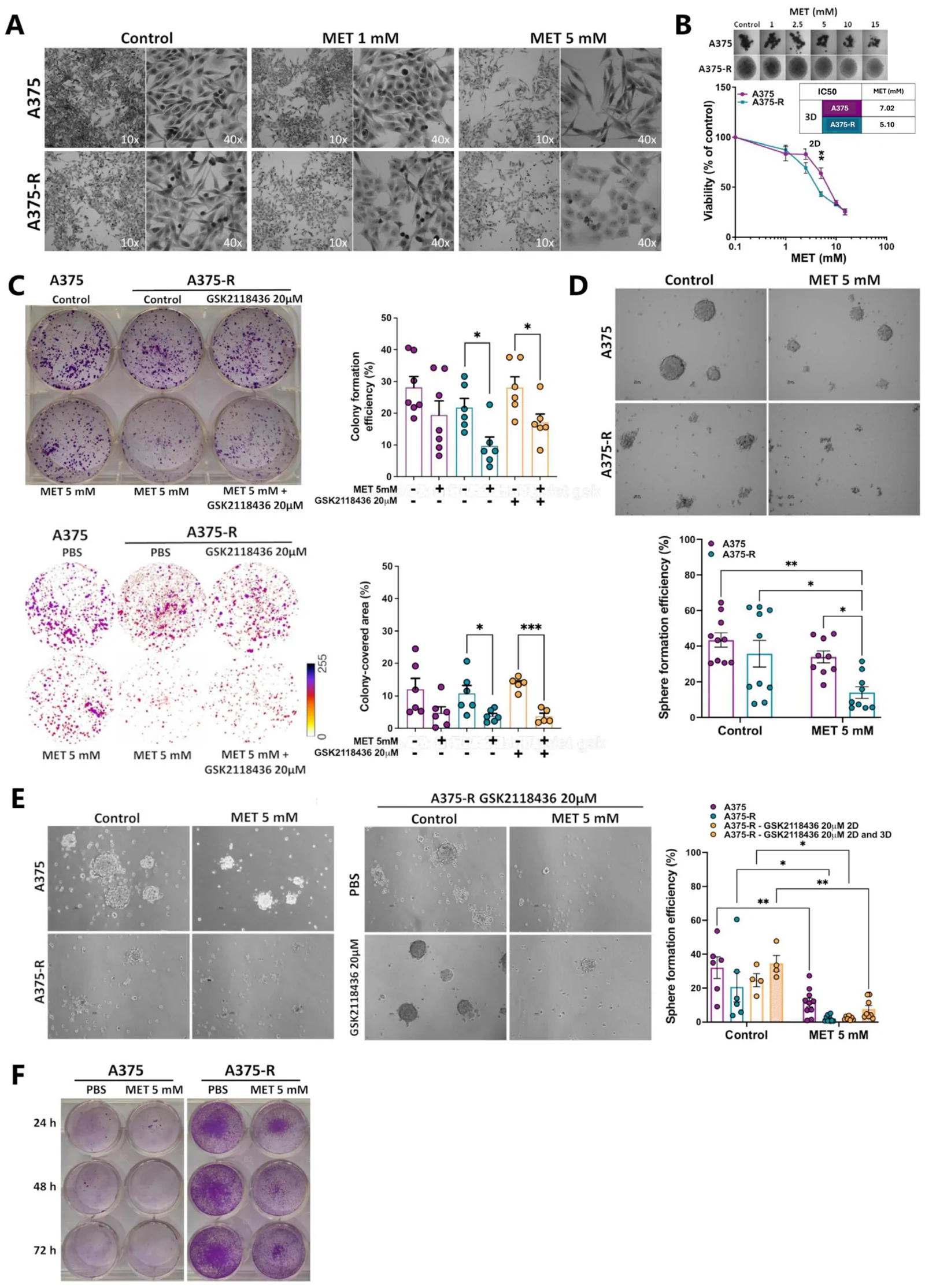
Cytotoxic effects of metformin in GSK2118436-resistant melanoma cells. A375 and A375-R cells were treated with metformin (MET; 1 or 5 mM) under 2D culture conditions and, after 48 h, stained with hematoxylin and eosin (**A**). Representative images illustrating morphological changes are shown (magnification: 100× and 400×). Spheroids cultured under 3D conditions were treated with increasing concentrations of MET (1–15 mM). After 10 days of treatment, cell viability was assessed using the APH assay (**B**). Results are expressed as viability relative to untreated controls (mean ± s.e.m.; *n* > 3). Data were analyzed by two-way ANOVA followed by Sidak’s post hoc multiple-comparison test. In addition, the colony-forming ability of A375 and A375-R cells was determined in the presence or absence of GSK2118436 (20 μM) and treated with MET (5 mM) (**C**). A representative image of the colonies obtained after 10 days using crystal violet staining is shown. Results are presented as colony-formation efficiency or colony-covered area calculated with ImageJ 1.52. At the bottom, the ImageJ quantification is shown; color intensity corresponds to the staining intensity of each pixel (mean ± s.e.m; *n* > 3; *t*-test). Sphere-forming capacity was also evaluated in cells treated or not with MET (5 mM) for 10 days (**D**). To this end, A375 and A375-R cells were analyzed using the residual sphere formation assay in the presence or absence of GSK2118436 (20 μM) and treated with MET (5 mM) under 2D conditions and then seeded under sphere-forming conditions (**E**). Representative images of spheres are shown (magnification: 100×). Results are presented as colony-formation efficiency (mean ± s.e.m; *n* > 3; two-way ANOVA with Sidak’s post hoc test). Cell viability of suspension cells previously treated with MET (5 mM) for 24, 48, or 72 h is shown (**F**). After 72 h of culture, cells were fixed and stained with crystal violet. Statistical significance: * *p* < 0.05, ** *p* < 0.01, *** *p* < 0.001.

For metformin, this effect was observed starting at 1 mM (*p* < 0.001) and was associated with a two-fold decrease in IC_50_, whereas for antimycin A, it was evident at 0.1 μg/mL (*p* < 0.001) and the IC_50_ decreased four-fold relative to A375 cells. Since metformin is a drug that has been widely tested and used clinically for more than 60 years, it was selected for further evaluation of OXPHOS inhibition. After metformin treatment, the morphology of the remaining A375-R cells differed from that observed in A375 cells (Figure 4A). At 1 mM metformin, clusters of cells with heterogeneous morphology were observed in both cell variants. However, at 5 mM metformin, polyhedral morphology was clear in the remaining A375-R cells, whereas A375 cells displayed a more spindle-shaped morphology. Subsequently, we assessed whether this effect was maintained under anchorage-independent conditions (3D). As observed under 2D conditions, A375-R cells were more sensitive to metformin than the parental cells (Figure 4B). This effect was evidenced by a leftward shift in the dose–response curve of A375-R cells (the IC_50_ of A375-R cells showed a 35% decrease compared to that of A375 cells), with a statistically significant difference at 5 mM metformin (*p* < 0.01).

Next, we decided to evaluate whether the cytotoxic effect of metformin could affect cells capable of forming colonies, i.e., those exhibiting stem-like characteristics and a lower division rate. To this end, clonogenic assays were performed in the presence or absence of the BRAFi GSK2118436 (20 μM) and then in the presence of 5 mM metformin. The colony-forming ability of the resistant cells was significantly affected by metformin in both the presence and absence of the BRAFi (Colony count: A375-R Control vs. MET *p* < 0.05 and A375-R GSK2118436 20 μM Control vs. MET *p* < 0.05; Colony area: A375-R Control vs. MET *p* < 0.05 and A375-R GSK2118436 20 μM Control vs. MET *p* < 0.001).

The effect of metformin on the sphere-forming capacity of the stem cell population was also evaluated. For this purpose, cells were seeded under sphere-forming conditions and treated with metformin (5 mM) for 10 days. The morphology of melanospheres formed by the resistant cells showed a clustered shape with irregular edges, low compactness, and easy disaggregation (Figure 4D). In contrast, A375 spheres were round, with smooth borders and high compactness (Figure 4D). Although no differences in sphere-forming capacity were observed between the cell lines, a significant difference in response to metformin was detected, with the resistant cell line being significantly more sensitive (*p* < 0.05).

To evaluate whether this effect of metformin could be long-lasting or “residual”, A375 and A375-R monolayers were next treated with metformin for 72 h in the presence or absence of GSK2118436 (20 μM) and then reseeded under sphere-forming conditions. A375-R cells in the presence of BRAFi were able to form spheres morphologically similar to A375 cells and showed a tendency toward a higher number of spheres. Previous metformin treatment drastically impaired the sphere-forming capacity in both cell lines. However, when the effect of metformin was specifically compared, the ability to generate melanospheres was significantly more affected in the resistant cell line than in the parental cells (*p* < 0.01). This effect was not reversed by co-incubation with metformin and the BRAFi under 2D conditions but showed a reduction in the residual effect of metformin when BRAFi was restored during sphere culture (*p* < 0.05).

The residual effect of metformin on the non-adherent population was also evaluated. For this purpose, cells were treated with metformin (5 mM) for 24, 48, or 72 h, the supernatant was collected, cells were separated by centrifugation, the drug-free medium was restored, and cells were cultured in a new plate for additional 72 h. Crystal violet staining allowed observing that the number of A375-R cells in the supernatant was indeed higher than that of A375 cells (Figure 4F). Furthermore, these cells were viable and capable of adhering to a new substrate to generate a monolayer. Metformin treatment impaired this adhesion capacity, as shown by comparison of the stained area in the metformin (5 mM) well with the control in A375-R cells at 24 h. This effect was more pronounced at 48 and 72 h.

**Figure 5:**
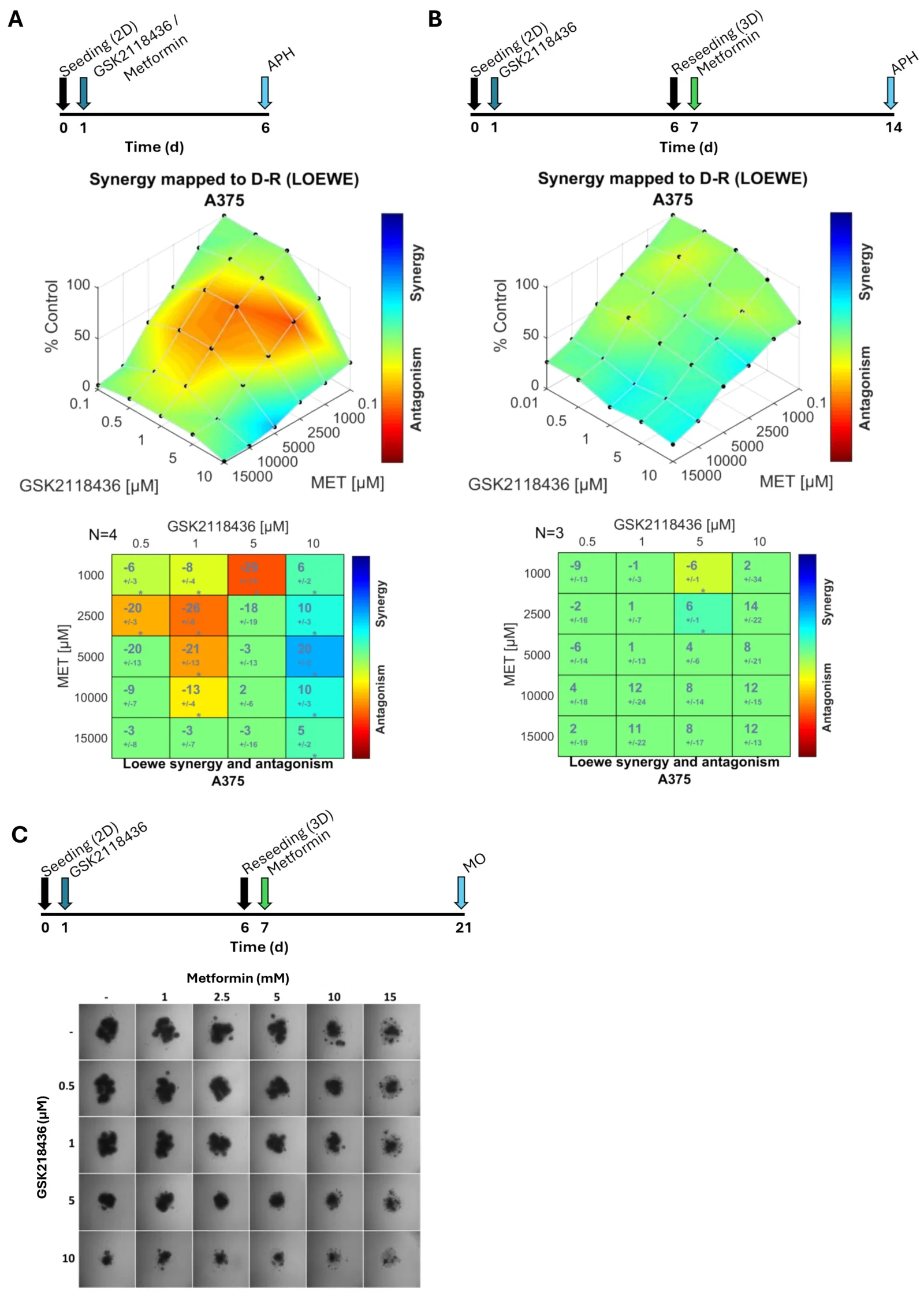
Cytotoxic effects of combined GSK2118436 and metformin treatment in melanoma cells. A375 cells were treated with increasing concentrations of the BRAF inhibitor GSK2118436 (0.5–10 μM) and metformin (MET; 1–15 mM), either simultaneously (**A**) or sequentially (**B**,**C**). After treatment, cell viability was assessed using the APH assay and spheroid morphology was evaluated by bright-field optical microscopy (MO). Surface-mapped analysis and significance matrices based on the Loewe additivity model were generated using Combenefit software 2.021 (**A**,**B**). Representative images of multicellular melanoma spheroids treated with BRAFi, MET, or their combination are shown (**C**) (magnification: 40×).

Finally, to determine whether the two drugs showed a synergistic, antagonistic, or additive effect, A375 cells were simultaneously exposed to increasing concentrations of GSK2118436 (0.5–10 μM) and metformin (1–15 mM). After 5 days of culture, cell viability was determined. Both the surface map analysis and the interaction matrix revealed small regions of synergy (light blue–blue) and additive effects (green) and broad regions of antagonism (yellow–orange). In particular, the antagonistic effect was evident at low concentrations of GSK2118436, where the effect of metformin as a single agent was greater than in combination with the BRAFi (*p* < 0.001; Figure 5A). Conversely, a synergistic effect was observed at higher concentrations of GSK2118436.

Based on these results and considering the previously observed effect of metformin on A375-R cells, we hypothesized that the acquisition of resistance to GSK2118436 might be required to improve the response to metformin.

Thus, we next designed a sequential treatment scheme in which A375 cells were first exposed to increasing concentrations of GSK2118436 (0.5–10 μM) and after 5 days of this treatment, the remaining cells were reseeded and treated with metformin (1–15 mM). After 5 days, cell viability was evaluated using the APH assay. Baseline analysis showed that the BRAFi response curve in the absence of metformin differed between both treatment schemes (Figure 5B). As in the simultaneous treatment, antagonistic and synergistic effects of the combination were evaluated using the Loewe model. Interestingly, in this sequential treatment scheme, no antagonistic regions (yellow areas) were observed in the surface map analysis, whereas an additive effect was observed throughout the concentration range (Figure 5B).

Given the limitations of long-term 2D cultures, the sequential treatment protocol was applied to A375 cells cultured under 3D conditions. In this model, the sequential treatment scheme was similar to that of the sequential 2D protocol: A375 cells were first exposed to increasing concentrations of GSK2118436 (0.5–10 μM) and after 5 days of this treatment, the remaining cells were reseeded in 3D conditions and treated with metformin (1–15 mM). After 5 days, spheroids decreased at high concentrations of both inhibitors compared to the respective single-agent treatments, particularly at metformin concentrations of 10–15 mM combined with GSK2118436 at 10 μM, thus reinforcing 2D results (Figure 5C).

## 3. Discussion

The development of resistance to BRAF inhibitors (BRAFi) remains a major clinical challenge in the treatment of BRAFV600E-mutant melanoma [21]. Although several genetic and signaling-based mechanisms have been described, increasing evidence indicates that non-genetic adaptations, including metabolic rewiring and phenotypic plasticity, play a critical role in the acquisition and maintenance of drug resistance. In the present study, we showed that melanoma cells with acquired resistance to the BRAFi GSK2118436 undergo profound phenotypic and metabolic changes that render them selectively vulnerable to mitochondrial metabolic inhibition with metformin. In this context, metformin-mediated complex I inhibition may selectively compromise the bioenergetic flexibility required for survival under MAPK pathway inhibition. Consistently, biguanides such as metformin have been shown to impair tumor growth and enhance the efficacy of targeted therapies in multiple preclinical models [10,11,22,23].

Recent preclinical studies have shown the capacity of metformin to sensitize tumor cells to therapy and to target metabolic dependencies associated with resistance. In melanoma and other cancer models, metformin has been shown to enhance the efficacy of MAPK pathway inhibitors [24,25], disrupt mitochondrial function [26] suppress mTOR signaling [27,28,29], and selectively target stem-like therapy-resistant cells [30,31,32]. Previous studies have also shown that metformin can modulate the tumor microenvironment and improve responses to immunotherapy in preclinical systems [33,34].

Our present data demonstrate that A375 melanoma cells that acquire resistance to the BRAFi GSK2118436 (A375-R) display marked morphological alterations and changes in the expression of epithelial–mesenchymal transition (EMT)-related markers such as vimentin, β-catenin, and E-cadherin. Resistant cells exhibited reduced E-cadherin expression in the non-adherent population, increased vimentin levels, and alterations in vimentin subcellular distribution, collectively suggesting a shift toward a mesenchymal-like phenotype. These findings are consistent with previous reports linking BRAFi resistance to EMT-like programs that promote cellular plasticity, invasiveness, and survival under therapeutic pressure [17].

Interestingly, N-cadherin expression did not uniformly increase in resistant cells, and, in some conditions, it was even reduced, highlighting the complexity and context dependency of cadherin switching in melanoma cells. This observation aligns with emerging concepts that, in melanoma, EMT does not necessarily follow a classical epithelial-to-mesenchymal trajectory but rather involves hybrid or partial EMT states that support drug tolerance and adaptive survival [35,36].

Moreover, the inability of A375-R cells to form compact spheroids under basal conditions, together with their recovery of spheroid formation upon BRAFi re-exposure, further supports the notion that resistance is accompanied by reversible phenotypic states rather than by fixed genetic alterations. Such plasticity may allow melanoma cells to dynamically adapt to changing microenvironmental and therapeutic cues.

A major finding of this study is that BRAFi-resistant melanoma cells exhibited a distinct metabolic vulnerability profile compared with their parental counterparts. Systematic screening of different metabolic modulators revealed that A375-R cells are significantly more sensitive to modulators targeting mitochondrial respiration, including metformin and antimycin A, and that their sensitivity to glycolytic inhibition or pyruvate redirection remained largely unchanged. These observations are in line with previously published studies that showed that the acquisition of BRAFi resistance is accompanied by a metabolic shift toward increased reliance on mitochondrial oxidative metabolism. Such a shift has been linked to enhanced mitochondrial biogenesis, increased OXPHOS activity, and altered redox homeostasis [37,38]. Our data reinforce this model and extend it by showing that mitochondrial vulnerability in resistant cells constitutes an exploitable therapeutic vulnerability.

In contrast, A375-R cells were less sensitive to modulators of folate metabolism and mTOR signaling, supporting the idea that resistant cells rewire multiple metabolic pathways to sustain survival. Notably, the reduced response to RAD001, an inhibitor of the mTOR pathway, is consistent with reports indicating that mTOR activation in BRAFi-resistant cells may become uncoupled from MAPK signaling and dependent on alternative pathways, including PI3K/AKT signaling or PTEN status [6,39,40].

Our data suggest that metformin effectively impairs the viability and reattachment capacity of cells present in the supernatant, a population often associated with anoikis resistance, stem-like properties, and metastatic potential. In addition, the strong effect of metformin on clonogenic and sphere-forming cells further suggests that this drug may preferentially target tumor-initiating or drug-tolerant persister populations that contribute to relapse.

In melanoma therapy, combination strategies aimed at preventing or overcoming resistance are of major interest. In this context, recent studies demonstrated a synergistic combination therapy of mitochondrion-targeting ligands and clinical inhibitors against melanoma [41]. Interestingly, we found that simultaneous treatment with BRAFi and metformin resulted predominantly in antagonistic effects, depending on the concentrations used. This observation is in agreement with previous reports showing heterogeneous and context-dependent outcomes of BRAFi–metformin combinations.

In contrast, a sequential treatment strategy—whereby melanoma cells were first exposed to BRAFi and subsequently treated with metformin—reduced antagonistic interactions and revealed mostly additive and synergistic effects at higher drug concentrations. Importantly, this sequential approach was also evidenced in 3D spheroid models, which better recapitulate tumor architecture and nutrient gradients.

From a translational perspective, our results support the concept that acquired BRAFi resistance creates a window of vulnerability to mitochondrial metabolic inhibitors such as metformin. Given the long-standing clinical use and favorable safety profile of metformin, these findings may have implications for treatment strategies in BRAFi-resistant melanoma patients. Nevertheless, this study has limitations, and in vivo validation will be required to confirm the therapeutic potential and safety of sequential BRAFi–metformin regimens. Despite this, early-phase clinical trials have demonstrated the safety and feasibility of combining metformin with anticancer therapies, reinforcing its translational potential as a low-cost, well-tolerated metabolic adjuvant [42,43]. In fact, at present, there is an ongoing phase I/II clinical trial of the BRAFi Vemurafenib and metformin in melanoma patients (NCT01638676) [44].

## 4. Materials and Methods

### 4.1. Cell Cultures

Human melanoma cell lines A375 (ATCC^®^ CRL-1619™) and A375-R were cultured at 37 °C in a humidified atmosphere of 95% air and 5% CO_2_ with DMEM/F12 medium (Invitrogen, Carlsbad, CA, USA) containing 10% FBS (Internegocios, Córdoba, Argentina), 10 mM HEPES (pH 7.4) and antibiotics (60 mg/L Penicillin G, 50 mg/L Streptomycin and 50 mg/L Gentamicin). For the 3D cultures, multicellular spheroids were obtained following the procedure of hanging drop culture [45] from trypsinized monolayers (0.8–1.4 × 10^4^ cell/spheroid). Mycoplasma contamination was routinely monitored by PCR throughout the study. The cell cultures were regularly tested to ensure that they remained free of mycoplasma contamination.

### 4.2. Determination of Cell Viability

To determine cell viability, cells were seeded onto 96-well plates at 4–7 × 10^3^ cells/well 24 h before treatments. After 5 days of treatments, cell viability was measured by the acidic phosphatase (APH) assay [46] and crystal violet staining [47].

### 4.3. MAPK Pathway Inhibitors

Increasing concentrations of the following BRAF and MEK inhibitors were evaluated: Dabrafenib (GSK2118436; Tafinlar^®^, GlaxoSmithKline, Brentford, UK), a selective BRAFV600E inhibitor with lower activity against BRAFV600R (PubChem CID: 44462760); Vemurafenib (PLX4032; Zelboraf^®^, Roche, Basel, Switzerland), a selective BRAFV600E inhibitor (PubChem CID: 42611257); Trametinib (GSK1120212; Mekinist^®^, Novartis, Basel, Switzerland), a MEK1/2 inhibitor (PubChem CID: 11707110); and PD98059 (Calbiochem, Merck, Darmstadt, Germany), a MEK1/2 inhibitor (PubChem CID: 4713).

### 4.4. BRAF Inhibitor–Resistant Melanoma Cell Line (A375-R)

The A375-R cell line was initiated by seeding A375 cells in T25 culture flasks and exposing them to increasing concentrations of the BRAFi GSK2118436. Treatment was started at 1 µM, based on the IC_50_ of parental A375 cells (0.88 µM), and the drug concentration was progressively increased during successive passages as cells recovered their proliferative capacity. In parallel, the parental A375 cell line was cultured under identical conditions but in the absence of the inhibitor and used as a control to discriminate drug-induced morphological and phenotypic changes from those associated with prolonged culture and passaging. After approximately four months of continuous drug selection, a stable resistant population (A375-R) was obtained in complete medium supplemented with 20 µM GSK2118436.

### 4.5. Determination of the Half-Maximal Inhibitory Concentration (IC_50_)

To determine the IC_50_, dose–response viability curves were generated for each cell line and treatment. The half-maximal inhibitory concentration (IC_50_) was calculated by fitting the data to a logarithmic inhibition curve with normalized response, using the GraphPad Prism software (version 8.0.1).

### 4.6. Evaluation of Synergistic or Antagonistic Drug Interactions

Drug combination effects were evaluated by using Combenefit 2.021 software according to the Loewe additivity model [48].

### 4.7. Expression of Epithelial–Mesenchymal Transition (EMT)-Related Markers

To evaluate the expression of EMT-related markers, including vimentin, β-catenin, and E-cadherin, adherent cells (harvested using 0.25 mM EDTA) and suspended cells from A375 and A375-R were first washed twice with cold PBS and permeabilized with 0.1% Triton X-100 for 10 min (vimentin determination only). Cells were then incubated with blocking solution (5% BSA in PBS) for 30 min, followed by incubation with primary antibodies diluted in blocking solution for 1 h at 4 °C. Excess primary antibody was removed by centrifugation (1500 rpm, 5 min, 4 °C), and cells were washed three times with cold PBS before incubation with the corresponding secondary antibody diluted in blocking solution for 2 h, protected from light. After incubation, cells were pelleted (1500 rpm, 5 min, 4 °C), resuspended in complete culture medium, and analyzed by flow cytometry to determine Alexa Fluor^®^488 fluorescence (FL1 channel), by using an Accuri^TM^ C6 Plus cytometer (BD, Biosciences, San Jose, CA, USA). Mean fluorescence intensity was determined and expressed relative to the geometric mean of A375 cells. The primary antibodies used were anti-E-cadherin (sc-8426, Santa Cruz Biotechnology, Dallas, TX, USA), anti-vimentin (ab92547, Abcam, Cambridge, UK), and anti-N-cadherin (13116S, Cell Signaling Technology, Danvers, MA, USA). The secondary antibodies used were Alexa Fluor^®^488-conjugated anti-rabbit IgG (ab150077, Abcam) and Alexa Fluor^®^488-conjugated anti-mouse IgG (ab150113, Abcam).

### 4.8. Metabolic Modulators

Increasing concentrations and/or combinations of the following metabolic modulators were evaluated: metformin (MET; Laboratorios Craveri, Buenos Aires, Argentina), a direct inhibitor of mitochondrial complex I and an indirect activator of AMP-activated protein kinase (AMPK) (PubChem CID: 4091); antimycin A (Sigma-Aldrich, St. Louis, MO, USA), a direct inhibitor of mitochondrial complex III (PubChem CID: 14957); 2-deoxy-D-glucose (Sigma-Aldrich, St. Louis, MO, USA), a glycolytic hexokinase inhibitor (PubChem CID: 108223); sodium oxamate (Sigma-Aldrich, St. Louis, MO, USA), a lactate dehydrogenase inhibitor (PubChem CID: 5242); 6-aminonicotinamide (Sigma-Aldrich), an inhibitor of glucose-6-phosphate dehydrogenase in the pentose phosphate pathway (PubChem CID: 9500); dichloroacetate (Merck, Darmstadt, Germany), an inhibitor of pyruvate dehydrogenase kinase (PubChem CID: 517326); methotrexate (Laboratorios Kampel Martian S.A, Buenos Aires, Argentina), a direct inhibitor of dihydrofolate reductase and modulator of mitochondrial metabolism (PubChem CID: 126941); N,N′-[thiobis(2,1-ethanediyl-1,3,4-thiadiazole-5,2-diyl)]bis-benzeneacetamide (BPTES; Cayman Chemical, Ann Arbor, MI, USA), a glutaminase inhibitor (PubChem CID: 3372016); and everolimus (RAD001; Novartis, Basel, Switzerland), an mTOR inhibitor acting through FKBP12 binding (PubChem CID: 6442177).

### 4.9. Western Blotting

Whole-cell extracts were obtained using a lysis and extraction buffer (50 mM tris-HCl (pH 8); 100 mM NaCl; 1% Triton; 10 mM EDTA; protease inhibitor 1:10,000). The lysates were centrifuged at 10,000 rpm for 10 min at 4 °C, and the supernatant was stored at −20 °C until immunoblotting was performed. Protein content was determined by the Bradford method. For the immunoblot, proteins (70–100 μg) from whole-cell extracts were electrophoresed on SDS-PAGE and transferred to PVDF membranes. The membranes were blocked with 5% nonfat milk for 1 h, incubated with the primary antibody overnight at 4 °C and exposed to the corresponding secondary antibody (1:5000) for 1 h at room temperature. The primary antibodies used were anti-E-cadherin (sc-8426, Santa Cruz Biotechnology), anti-vimentin (ab92547, Abcam), anti-N-cadherin (13116S, Cell Signaling Technology) and anti-beta tubulin (16308, Invitrogen). Densitometry units were referred to tubulin. The secondary antibodies used were goat anti-rabbit IgG-HRP (Sigma A9169) and goat anti-mouse IgG-HRP (Santa Cruz sc-2031). Chemiluminescence was detected using the Image Quant LAS 500 (GE Healthcare Life Sciences, Uppsala, Sweden).

### 4.10. Statistical Analysis

All experiments were performed at least in triplicate, and data are expressed as the mean ± s.e.m. Differences between groups were analyzed with one- or two-way ANOVA followed by multiple comparisons Tukey’s test. *p* < 0.05 was established as significant. Analyses were made using INFOSTAT free edition and GraphPad Prism 8.0.1 software (GraphPad Software Inc., USA, San Diego, CA, USA).

## 5. Conclusions

In the present study, we established an A375 melanoma cell subline with acquired BRAFi resistance that displayed cross-resistance to other MAPK inhibitors and phenotypic changes associated with EMT. Importantly, metabolic drug screening revealed a selective vulnerability of A375-R cells to mitochondrial inhibition, as they were significantly more sensitive to metformin and antimycin A than parental A375 cells. Metformin also markedly impaired the clonogenic and sphere-forming capacities of resistant cells, supporting a functional impact on tumorigenic properties.

Notably, the response to BRAFi and metformin was schedule-dependent. While simultaneous treatment was predominantly antagonistic, sequential treatment with BRAFi followed by metformin reduced antagonism and resulted in synergistic effects at higher concentrations. Overall, these findings indicate that acquired BRAFi resistance is associated with a metabolic adaptation that increases melanoma cell vulnerability to mitochondrial targeting and support further investigation of sequential MAPK and metabolic inhibition as a potential strategy for BRAFi-resistant melanoma.

## Figures and Tables

**Table 1:** Inhibitory concentrations of metabolic modulators in melanoma cells.

	2DG (mM)	OXA (mM)	6AN (μM)	MET (mM)	ANT (μg/mL)	DCA (mM)	MTX (nM)	BPTES (mM)	RAD (nM)
A375	>10	84.29	>100	4.22	0.002	16.89	734	>10	>100
A375R	>10	59.39	>100	2.35	0.005	17.94	>1000	>10	>100

Table 1 shows the inhibitory concentrations corresponding to 50% cell viability (IC_50_) for 2-deoxyglucose (2-DG), oxamate (OXA), 6-aminonicotinamide (6-AN), metformin (MET), antimycin A (ANT), dichloroacetate (DCA), methotrexate (MTX), N,N′-[thiobis(2,1-ethanediyl-1,3,4-thiadiazole-5,2-diyl)]bis-benzeneacetamide (BPTES), and everolimus (RAD001) in human melanoma cells cultured under 2D conditions. Cell viability data were fitted to a log(inhibitor) versus normalized response curve. IC_50_ values were obtained from the analysis of the fitted curves using GraphPad Prism 8.0.1.

## Data Availability

The original contributions presented in this study are included in the article. Further inquiries can be directed to the corresponding author.
